# Genetic characterization of African swine fever virus isolates from soft ticks at the wildlife/domestic interface in Mozambique and identification of a novel genotype

**DOI:** 10.1111/tbed.12700

**Published:** 2017-09-17

**Authors:** C. J. Quembo, F. Jori, W. Vosloo, L. Heath

**Affiliations:** ^1^ Transboundary Animal Disease Program Onderstepoort Veterinary Institute Pretoria South Africa; ^2^ Department of Veterinary Tropical Diseases Faculty of Veterinary Sciences University of Pretoria Pretoria South Africa; ^3^ Laboratório Regional de Veterinária em Chimoio Centro Zonal de Investigação Agrária da Zona Centro Instituto de Investigação Agrária de Moçambique Chimoio Mozambique; ^4^ UMR ASTRE CIRAD Montpellier France; ^5^ Department of Zoology & Entomology Mammal Research Institute University of Pretoria Pretoria South Africa; ^6^ Australian Animal Health Laboratory Geelong Vic. Australia

**Keywords:** African swine fever, genotype, Mozambique, *Ornithodoros moubata*, virus, warthog

## Abstract

African swine fever virus (ASFV) is one of the most threatening infectious diseases of pigs. There are not sufficient data to indicate the importance of the sylvatic cycle in the spread and maintenance of the disease locally and potentially, globally. To assess the capacity to maintain ASF in the environment, we investigated the presence of soft tickreservoirs of ASFV in Gorongosa National Park (GNP) and its surrounding villages. A total of 1,658 soft ticks were recovered from warthog burrows and pig pens at the wildlife/livestock interface of the GNP and viral DNA was confirmed by nested PCR in 19% of *Ornithodoros porcinus porcinus* and 15% of *O. p. domesticus*. However, isolation of ASFV was only achieved in approximately 50% of the PCR‐positive samples with nineteen haemadsorbing virus isolates recovered. These were genotyped using a combination of partial sequencing of the B646L gene (*p72*) and analysis of the central variable region (CVR) of the B602L gene. Eleven isolates were classified as belonging to genotype II and homologous to contemporary isolates from southern Africa, the Indian Ocean and eastern Europe. Three isolates grouped within genotype V and were similar to previous isolates from Mozambique and Malawi. The remaining five isolates constituted a new, previously unidentified genotype, designated genotype XXIV. This work confirms for the first time that the virus currently circulating in eastern Europe is likely to have a wildlife origin, and that the large diversity of ASFV maintained in wildlife areas can act as a permanent sources of different strains for the domestic pig value chain in Mozambique and beyond its boundaries. Their genetic similarity to ASFV strains currently spreading across Europe justifies the need to continue studying the sylvatic cycle in this African country and other parts of southern Africa in order to identify potential hot spots of ASF emergence and target surveillance and control efforts.

## 1 INTRODUCTION

African swine fever virus (ASFV), the only member of the Asfarviridae family, often leads to high mortalities in domestic pigs (Penrith, Vosloo, Jori, & Bastos, 2013), resulting in devastating impacts in the pig industry of many countries in sub‐Saharan Africa, the Indian Ocean and more recently in eastern Europe (Costard et al., 2009; Gallardo et al., 2014).

In East and southern Africa, this highly infectious virus is maintained through a sylvatic cycle involving warthogs (*Phacochoerus africanus)* and soft ticks of the *Ornithodoros moubata* complex (Jori & Bastos, 2009). Ticks become infected with the virus when they feed on infected warthogs. They are able to maintain the infection across successive generations of soft ticks by transovarial, venereal and trans‐stadial transmission (Kleiboeker, Burrage, Scoles, Fish, & Rock, 1998; Rennie, Wilkinson, & Mellor, 2001), facilitating maintenance of the ASFV within soft tick population for a period of at least 15 months (Plowright, Thomson, & Neser, 1994). Additionally, in Madagascar, the presence of ASFV in ticks in unoccupied domestic pig premises (for at least 4 years) has been found. Thus, these vectors are able to maintain the infection for long periods (Ravaomanana et al., 2010).

No consistent data are available regarding the occurrence of a sylvatic cycle for many other areas of Africa. This is the case for several countries in West or Central Africa (Jori & Bastos, 2009; Jori et al., 2007, 2013; Penrith et al., 2004) except Senegal where *Ornithodoros sonrai* has been identified in pig pens and rodent burrows (Jori et al., 2013; Vial et al., 2006, 2007). The active search in warthog burrows in Senegal and other West African countries failed to demonstrate the presence of Ornithodoros ticks (Vial et al., 2007).

The boundaries of protected areas in East and southern Africa, where large populations of warthogs and *O*. *moubata* ticks cohabitate, represent key areas for ASFV dissemination from wild hosts into the domestic pig value chain (Jori & Bastos, 2009; Jori et al., 2007, 2013). The most common transmission pathway of ASFV from sylvatic to domestic hosts is suspected to be through infected ticks feeding on domestic pigs at the wildlife/livestock interface. The ASF sylvatic cycle has been described in detail in many countries in East and southern Africa (Okoth et al., 2013).

In Mozambique, serological evidence of ASFV and soft ticks has been reported in recent years at the wildlife/livestock interface of the Gorongosa National Park (GNP). In addition, this area included several risk factors known to be associated with the occurrence and dissemination of ASF infection such as the presence of free‐ranging pigs, the absence of biosecurity measures and the trade of live pigs and pork products with other major pig production and consumption areas in the country (Penrith et al., 2007; Quembo, Jori, Heath, Pérez‐Sánchez, & Vosloo, 2014). However, no information has been published, to date, on the presence or the genetic characteristics of ASFV maintained in wild hosts in any Mozambican wildlife area.

African swine fever virus is the only double‐stranded DNA‐containing arbovirus (dsDNA) with a genome of 170–193 kilobase pairs (kbp) (Dixon, Chapman, Netherton, & Upton, 2013). Four different gene regions (*p72, p30, p54* and *B602L)* have been targeted to detect ASFV phylogenetic relationships with different levels of precision (Bastos, Penrith, Macome, Pinto, & Thomson, 2004; Gallardo et al., 2009, 2011; Nix, Gallardo, Hutchings, Blanco, & Dixon, 2006). Molecular analysis of ASFV strains worldwide indicated that genetic homogeneity within genotypes appears more frequently in areas without the involvement of any sylvatic cycle (Bastos et al., 2003, 2004; Lubisi, Bastos, Dwarka, & Vosloo, 2007). Conversely, in certain endemic areas of southern and eastern Africa where the warthog–tick sylvatic cycle occurs, the genetic diversity of ASFV circulating strains has been reported to be higher (Bastos, Arnot, Jacquier, & Maree, 2009; Penrith et al., 2013).

Therefore, the aims of this investigation were to assess the presence of ASFV‐infected soft ticks at the wildlife/livestock interface of the GNP and to characterize the genetic diversity of the viruses found in soft ticks and compare to ASFV isolates found elsewhere in Africa or Europe.

## 2 MATERIALS AND METHODS

### 2.1 Study area

The field work was carried out in the Gorongosa District (GD), Province of Sofala, Central Mozambique (latitude 18°45′/19°15′ South and longitude 33°30′/34°45′ East), that covers a total area of 7,659 km² with approximately 92,550 habitants in 19 villages. At least 30% of its inhabitants are small‐scale farmers, which mainly rely on subsistence agricultural activities as a source of income (District Agricultural Directorate, unpublished report of 2005).

Half of the GD surface is occupied by the GNP, which covers a total area of 3,770 km^2^ represented by a diversity of habitats including African plains, rivers and lakes and mountain chains reaching 1,860 m above sea level. Average rainfall varies from 900 to 2,000 mm per year, mostly during November and April, and the normal temperature varies from 25 to 33°C.

The wildlife/domestic interface at the eastern edge of the GNP includes seven villages located <10 km from the GNP where interactions between domestic pigs and wild hosts are considered common, and is referred to as the buffer zone (BZ). The pig production systems in this area are characterized by basic small‐scale pigsties where pigs are left to roam freely for most of the year, with the exception of the cropping season. This area has experienced several suspected outbreaks of ASF since 2005, although none of them was confirmed by laboratory testing (Penrith et al., 2007; Quembo et al., 2014).

### 2.2 Soft ticks sampling protocol

**Figure 1:**
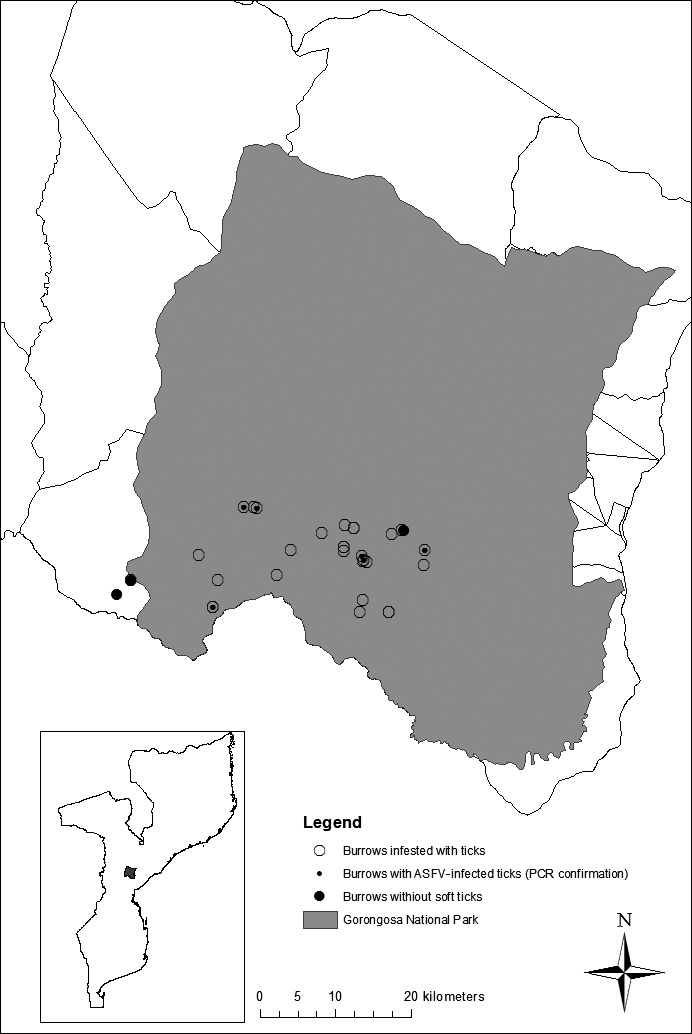
Map showing the locations of the warthog burrows inspected, indicating those infested with ticks, those where soft tick presence could not be detected, and those in which African swine fever virus presence was confirmed by PCR

Tick sampling in the GNP was carried out in warthog burrows. Sample size was determined based on an estimated population of 4,000 warthogs (C. L. Pereira, personal communication 2005), an average size of four individuals per warthog sounder and an average of three burrows used by sounder (Estes, 2008). The estimated numbers of burrows was expected to be at least 3,000. The expected prevalence of warthog burrows infested with *O. moubata* soft ticks was estimated between 30% and 88% on the basis of previous reports from areas in East and southern Africa (Plowright et al., 1994). Using Win Episcope 2.0 (Thrusfield, Ortega, de Blas, Noordhuizen, & Frankena, 2001), the sample size to detect at least one infested burrow among a total of 3000, considering a minimum soft tick infestation rate of 30% and a 95% level of confidence, was estimated to be a minimum of nine burrows. To ensure the probability of detection in case the local infestation prevalence was lower than 30%, we inspected a total of 32 warthog burrows distributed within an area of 400 km^2^ in the south‐eastern region of the GNP (Figure 1).

The sampling frame and the characteristics of domestic pig farms were the same as those described in a previous work (Quembo et al., 2014). Due to logistic constraints and limited resources, only convenience sampling for soft ticks was conducted in 20 domestic pig pens distributed in 18 villages from GD between March 2006 and September 2007. Four of these villages are located in the BZ, while the remaining seven sampled villages were located further in the district.

A total of 1,662 soft ticks were collected from the warthog burrows by removing loose sand and litter manually using a spade, followed by vacuum aspiration to collect material from cracks in the walls and lining of the burrows. Soft ticks were manually extracted in the domestic pig pens from accessible crevices, small holes and external structures, whereas deeper fissures were examined using a portable petrol‐powered vacuum aspirator (Jori et al., 2013; Ravaomanana et al., 2010). Collections from warthog burrows and domestic pig pens occurred during the same period (March 2006–September 2007). The ticks were placed in 10‐ml‐labelled containers filled with sand and covered with a perforated lid. These specimens were dispatched live under permit to the Transboundary Animal Disease Programme (TADP), ARC‐Onderstepoort Veterinary Institute (OVI) in South Africa for further analysis.

### 2.3 Soft tick sample classification

The soft ticks were sorted into different categories depending on their stage of development (Table 1) using standard methods (Walton, 1962, 1979). Adult soft ticks (males and females) were tested in pools of three per habitat (warthog burrow or pig pen), while the immature stages (N1–N5) were pooled in groups of maximum 6 per developmental stage and habitat. Pools of all eggs collected from each sampling point were tested (Table 1). Each pool was crushed in 1 ml of phosphate‐buffered saline supplemented with 1% foetal calf serum and 1% of a combination of antibiotics and antimycotics. The homogenates were clarified by centrifugation at 10,000 *g* for 1 min and the supernatants stored at −70°C.

**Table 1:** Observed prevalence of African swine fever virus (ASFV) in different development stages of *Ornithodoros* spp. found inside the Gorongosa National Park and in the buffer zone surrounding the park

Gorongosa National Park					Buffer zone			
Development stage	No. of soft ticks collected	No. of pools of tested soft ticks	No. and (%) positives for nested PCR^a^	No. and (%) of ASFV isolation^b^	No. of soft ticks collected	No. of pools of tested soft ticks	No. and (%) positives for nested PCR^a^	No. and (%) of ASFV isolation^b^
Male	115	61	4 (6.6)	2 (50.0)	6	3	0	0
Female	130	52	10 (19.2)	5 (50.0)	9	3	0	0
N1	26	9	4 (44.4)	2 (50.0)	0	0	0	0
N2	78	16	2 (12.5)	1 (50.0)	31	3	1 (33.3)	0
N3	250	41	14 (34.1)	4 (28.6)	14	2	1 (50.0)	1 (100)
N4	353	43	13 (30.2)	7 (53.8)	62	7	2 (28.6)	1 (50.0)
N5	710	77	8 (10.4)	5 (62.5)	81	8	0	0
Eggs	Not counted^c^	5	2 (40.0)	1 (50.0)	0	0	0	0
Total	1,662	304	57 (18.8)	27 (47.4)	203	26	4 (15.4)	2 (50.0)

a Percentage and number of tested pools positive for ASFV DNA using the nested PCR.

b Percentage of positive isolations was calculated in relation to the number of individuals or pools of ticks testing positive with the nested PCR.

c The soft tick eggs were too numerous to count.

### 2.4 Virus isolation

African swine fever viruses were isolated from soft ticks using a haemadsorption assay previously described (Malmquist & Hay, 1960). One hundred microlitres of the supernatant was inoculated onto peripheral blood mononuclear cells (PBMC) and cultured following standard procedures (Malmquist & Hay, 1960). All samples were subjected to three blind serial passages on PBMC.

### 2.5 DNA extraction and PCR

DNA was extracted from 200 μl of cell culture supernatant using a commercial kit (Qiagen GmbH, Hilden, Germany) or from 200 μl aliquots of tick homogenates following a silica/guanidium‐based nucleic acid extraction protocol (Boom et al., 1990) and stored at −80°C.

A nested PCR was used to screen soft tick samples for the presence of ASFV DNA as well as the tick 16S rDNA as previously published (Basto et al., 2006; Ravaomanana et al., 2010). A pig farm or a warthog burrow was considered positive to ASFV when at least one soft tick sample was found positive.

Positive samples were subsequently used to amplify various gene regions (*p72* (Bastos et al., 2003), *p30, p54* (Gallardo et al., 2009, 2011) and *B206L* (Nix et al., 2006)) for nucleotide sequencing. PCR products were excised from a 1% agarose gel and purified using the NucleoSpin Extract Kit from Macherey‐Nagel, GmbH & Co. KG (Düren, Germany).

### 2.6 DNA sequence and phylogenetic analysis

The nucleotide sequences of the purified products were obtained by automated sequencing at Inqaba biotech™ (Pretoria, South Africa) using the Big Dye terminator kit (Applied Biosystems, USA) and the same primer sets used for amplification.

Nucleotide sequences were edited and aligned using Sequencher™ Gene Codes Version 4.8 (Gene codes Corporation, MI, USA) and Mega 7.0 (Kumar, Stecher, & Tamura, 2007) software packages, respectively. The Kimura 2‐parameter model was selected as the best‐fit model using ModelTest (Posada & Crandall, 1998) implemented in Mega 7.0 (Kumar et al., 2007). Neighbour‐joining (NJ) and Maximum likelihood phylogenetic trees were constructed using Mega 7.0 (Kumar et al., 2007) with nodal support being assessed by 1,000 bootstrap replicates. Bayesian inference was conducted using MrBayes v3.2.6, running four chains simultaneously using default settings (Huelsenbeck & Ronquit, 2001). Thirty‐five ASFV isolates representative of 23 genotypes isolated in several countries from sub‐Saharan Africa (Achenbach et al., 2016; Bastos et al., 2004), the Indian Ocean (Lubisi, Dwarka, Meenowa, & Jaumally, 2009) and Europe (Gallardo et al., 2014) were included in the analysis (Table 2). The *B602L* gene region was also used to compare different isolates (Nix et al., 2006). The gaps, shown in Table 3, were inserted for alignment purposes and were executed in Mega 7.0 analysis software (Kumar et al., 2007).

**Table 2:** List of the 55 ASFV isolates from different locations and those obtained in this study from soft ticks inside the GNP and in its buffer zone

Virus name	Place/Country of origin	Host species	*P72* Genbank accession no.	Genotype	References	*CVR* GenBank accession no.	References
Mozambique/1960	Tete/Mozambique	Domestic pig	AF270708	V	Bastos et al. (2004)	AY274465	Bastos et al. (2004)
Tengani/60	Tengani/Malawi	Warthog	AF301541	V	Bastos et al. (2003)	AY538727	Bastos et al. (2004)
MAL 1/02	Mpemba/Malawi	Domestic pig	AY494553	V	Lubisi et al. (2005)	N/A	
Mozambique/1979	Beira/Mozambique	Domestic pig	AF270709	V	Bastos et al. (2004)	AY274470	Bastos et al. (2004)
SPEC/265	Maputo/Mozambique	Domestic pig	AF270710	VI	Bastos et al. (2003)	AY274467	Bastos et al. (2004)
MOZ 8/1994	Manica/Mozambique	Domestic pig	AF270712	VI	Bastos et al. (2004)	AY274469	Bastos et al. (2004)
MOZ 1/1994	Maputo/Mozambique	Domestic pig	AF270711	VI	Bastos et al. (2003)	AY274468	Bastos et al. (2004)
Lillie	South Africa	Domestic pig	DQ250109	XX	Boshoff et al. (2007)	DQ250109	Boshoff et al. (2007)
RSA 1/1999	Thabazimbi/South Africa	Warthog	AF302818	IV	Bastos et al. (2003)	DQ250108	Boshoff et al. (2007)
RSA 1/98	Potgietersrus/South Africa	Domestic pig	AF302818	VII	Bastos et al. (2003)	DQ250106	Boshoff et al. (2007)
MAD 1/1998	Madagascar	Domestic pig	AF270706	II	Bastos et al. (2003)	AY274471	Gonzague et al. (2001)
MAU 1/2007	Mauritius	Domestic pig	FJ528594	II	Lubisi et al. (2009)	N/A	J. Van Heerden, and L. Heath, Unpublished
MOZ 2/2002	Nampula/Mozambique	Domestic pig	AY351518	II	Lubisi et al. (2005)	N/A	J. Van Heerden, and L. Heath, Unpublished
ZIM 1/1992	Gweru/Zimbabwe	Domestic pig	DQ250119	XVII	Boshoff et al. (2007)	DQ250097	Boshoff et al. (2007)
Lisbon/60	Lisbon/Portugal	Domestic pig	AF301539	I	Bastos et al. (2003)	AM259405	Nix et al. (2006)
BEN/1/1997	Benin	Domestic pig	AF302816	I	Bastos et al. (2003)	AM712239	Chapman et al. (2008)
ANG/70	Angola	Domestic pig	AF301542	I	Bastos et al. (2003)	AM259411	Nix et al. (2006)
SPEC/205	Windhoek/Namibia	Domestic pig	DQ250114	I	Boshoff et al. (2007)	DQ250092	Boshoff et al. (2007)
NYA/12	Kalumo/Zambia	Ticks	AY351555	XIV	Lubisi et al. (2005)	N/A	
TAN 2/2003	Arusha/Tanzania	Domestic pig	AY494551	XVI	Lubisi et al. (2005)	N/A	
TAN 1/2001	Tanzania	Domestic pig	AY494552	XV	Lubisi et al. (2005)	N/A	
SUM/1411	Sumbu Park/Zambia	Ticks	AY351542	XIII	Lubisi et al. (2005)	N/A	
MZI/1/92	Mzinda/Malawi	Domestic pig	AY351543	XII	Lubisi et al. (2005)	N/A	
KAB/62	Livingstone/Zambia	Ticks	AY351522	XI	Lubisi et al. (2005)	N/A	
SPEC/257	Ellisras/South Africa	N/K	DQ250120	III	Boshoff et al. (2007)	DQ250098	Boshoff et al. (2007)
RSA 3/1996	Pienaarsrivier/South Africa	N/K	DQ250127	XIX	Boshoff et al. (2007)	DQ250105	Boshoff et al. (2007)
RSA 1/1996	Gravelotte/South Africa	N/K	DQ250125	XXI	Boshoff et al. (2007)	DQ250103	Boshoff et al. (2007)
SPEC/245	Louis Trichardt/South Africa	N/K	DQ250117	XXII	Boshoff et al. (2007)	DQ250095	Boshoff et al. (2007)
NAM 1/1995	Windhoek, Namibia	N/K	DQ250122	XVIII	Boshoff et al. (2007)	DQ250100	Boshoff et al. (2007)
MOZ 1/1998	Tete/Mozambique	Domestic pig	AF270705	VIII	Bastos et al. (2003)	N/A	OVI (J. Van Heerden, and L. Heath, unpublished)
BUR 1/1984	Burundi	Domestic pig	AF449463	X	Bastos et al. (2003)	AM259422	Nix et al. (2006)
UGA 1/2003	Uganda	Domestic pig	AY351564	IX	Lubisi et al. (2005)	AM259420	Nix et al. (2006)
UGA 1/1995	Mulindue/Uganda	Domestic pig	AF449475	IX	Bastos et al. (2003)	N/A	
ETH/3	Debre Zeit farm/Ethiopia	Domestic pig	KT795360	XXIII	Achenbach et al. (2016)	KT795372	Achenbach et al. (2016)
ETH/1504	Debre Zeit farm/Ethiopia	Domestic pig	KU291454	XXIII	Achenbach et al. (2016)	KU291452	Achenbach et al. (2016)
GEO 1/2007	Georgia	Domestic pig	AM999764	II	Rowlands et al. (2008)	AM999767	Rowlands et al. (2008)
Moz 1/2006	Gorongosa National Park	Soft tick (N2)	KY353980	II	This study	KY353964	This study
Moz 2/2006	Gorongosa National Park	Soft tick (N4)	KY353981	II	This study	KY353965	This study
Moz 3/2006	Gorongosa National Park	Soft tick (N5)	KY353982	II	This study	KY353966	This study
Moz 4/2006	Gorongosa National Park	Soft tick (N5)	KY353983	II	This study	KY353967	This study
Moz 5/2006	Gorongosa National Park	Soft tick (N1)	KY353984	II	This study	KY353968	This study
Moz 6/2006	Gorongosa National Park	Soft tick (N4)	KY353985	II	This study	N/A	
Moz 7/2006	Gorongosa National Park	Soft tick (N5)	KY353986	II	This study	KY353969	This study
Moz 8/2006	Gorongosa National Park	Soft tick (N5)	KY353987	II	This study	KY353971	This study
Moz 9/2006	Gorongosa National Park	Soft tick (Eggs)	KY353988	II	This study	KY353972	This study
Moz 10/2006	Gorongosa National Park	Soft tick (Male)	KY353989	XXIV	This study	KY353978	This study
Moz 11/2006	Gorongosa National Park	Soft tick (N4)	KY353990	XXIV	This study	N/A	
Moz 12/2006	Gorongosa National Park	Soft tick (N4)	KY353991	II	This study	KY353970	This study
Moz 13/2006	Gorongosa National Park	Soft tick (Female)	KY353992	II	This study	KY353973	This study
Moz 14/2006	Gorongosa National Park	Soft tick (Female)	KY353993	V	This study	KY353975	This study
Moz 15/2006	Gorongosa National Park	Soft tick (N4)	KY353994	V	This study	KY353977	This study
Moz 16/2006	Gorongosa National Park	Soft tick (N4)	KY353995	XXIV	This study	KY353976	This study
Moz 17/2006	Gorongosa National Park	Soft tick (N3)	KY353996	V	This study	KY353974	This study
Moz 18/2006	GNP buffer zone	Soft tick (N3)	KY353997	XXIV	This study	KY353979	This study
Moz 19/2006	GNP buffer zone	Soft tick (N4)	KY353998	XXIV	This study	N/A	

ASFV = African swine fever virus; GNP = Gorongosa National Park; N/A** **= not available.

**Table 3:** Central variable regions (CVR) of the 9RL ORF tetrameric amino acid repeat alignment from the isolates collected in the Gorongosa National Park and buffer zone

Isolate	Place/country of origin	Species of origin	P72 Genotype	CVR tetrameric amino acids sequences	No. of repeats	CVR subgroups	Reference
**Moz 2/2006**	Gorongosa Park	Ticks	II	BNDBNDBNAL	10	XXXII	This study
**Moz 3/2006**	Gorongosa Park	Ticks	II				This study
**Moz 4/2006**	Gorongosa Park	Ticks	II				This study
**Moz 5/2006**	Gorongosa Park	Ticks	II				This study
**Moz 7/2006**	Gorongosa Park	Ticks	II				This study
**Moz 8/2006**	Gorongosa Park	Ticks	II				This study
**Moz 9/2006**	Gorongosa Park	Ticks	II				This study
**Moz 12/2006**	Gorongosa Park	Ticks	II				This study
**Moz 13/2006**	Gorongosa Park	Ticks	II				This study
MAD/1/98	Madacascar	Domestic pig	II				Bastos et al. (2004)
MAU/2007/1	Mauritius	Domestic pig	II				OVI (J. Van Heerden, and L. Heath, unpublished)
MOZ/2/02	Mozambique	Domestic pig	II				OVI (J. Van Heerden, and L. Heath, unpublished)
Tengani/60	Tengani/Malawi	Warthog	V	ABABNBABHAL	11	XXI	Nix et al. (2006)
**Moz 1/2006**	Gorongosa Park	Ticks	II	APSPSOPNAFNOFFNFOPNAFNOFFNQVQMV	31	XXIa	This study
**Moz 15/2006**	Gorongosa Park	Ticks	V				This study
**Moz 14/2006**	Gorongosa Park	Ticks	V	ABHABNBABHAL	12	XXIb	This study
**Moz 17/2006**	Gorongosa Park	Ticks	V				This study
Moz/1979	Beira/Mozambique	Domestic pig	V				Bastos et al. (2004)
Moz/1960	Tete/Mozambique	Domestic pig	V	ABNAAAALBNBNBABNBABHAL	22	XXIc	Bastos et al. (2004)
**Moz 10/2006**	Gorongosa Park	Ticks	XXIV	BNAABNBNA	9	XXXIII	This study
**Moz 16/2006**	Gorongosa Park	Ticks	XXIV	ABHAABNBBHAL	12	XXXIIIa	This study
**Moz 18/2006**	Gorongosa District	Ticks	XXIV	ABTAAAACBNAAAAACBNAAAAACKTAAAACBNAKA	36	XXXIIIb	This study

The single letters refers to the code of each tetrameric repeat: B = CADT; N = NVDT/NVGT; D = CASM; A = CAST; L = CTST; H = NEDT; P = NADT; S = SAST; O = NASI; F = NAST; Q = NADI; V = NANT; M = NANI; T = NVNT; C = GAST; K = CANT (Bastos et al., 2004; Boshoff et al., 2007; Chapman et al.*,* 2008; Nix et al., 2006).

The isolates from this study are marked in bold.

## 3 RESULTS

### 3.1 Soft tick collection

A total of 1,865 argasid ticks were collected, 89.1% being found in warthog burrows from the south‐east region of the GNP and 10.9% in pig pens from its BZ. The apparent prevalence of infestation in the burrows (*n* = 32) was 90.6%, 95% CI (82.2–99.0), while argasid ticks were only found in 10% (2/20), 95% CI (3.1–23.1) of the pig pens inspected.

The proportions of tick specimens collected by zone (GNP and BZ), sex and stage of development and location are shown in Table 1. The majority (89.1%)—corresponding to the ticks collected in burrows—was morphologically identified as *Ornithodoros porcinus porcinus*, while only 10.9% —corresponding to the tick specimens found in pig pens—was classified as *Ornithodoros porcinus domesticus*. The classification was confirmed by means of 16S rDNA gene sequencing (data not shown).

The most predominant tick stages collected (all sites) were immature or nymph specimens (N1–N5) with N5 most abundant. Female soft ticks were the most abundant among the adults (Table 1). Additionally, 15 hard ticks of the genera *Amblyomma*,* Haemaphysalis* and *Rhipicephalus* were found only in burrows, but were not included for viral analysis.

### 3.2 Analysis of ASFV infection in collected soft ticks

African swine fever virus DNA was detected by PCR in 72.4%, 95% CI (52.8–92.0) of the soft ticks collected in burrows (18.8%, 95% CI [14.4–23.1] of the 304 pooled samples) and 15.4%, 95% CI (1.53–29.3) of the 26 pooled soft tick specimens collected in pigsties (Table 1). A total of 29 ASF viruses were isolated from 61 pooled samples representing a virus recovery rate of 47.4%.

### 3.3 *p72* Gene relationships between ASFV from soft ticks in different habitats

To classify the 29 virus isolates obtained from soft ticks at the GNP into the previously described major ASFV genotypes, the C‐terminal of the p72 gene was amplified and sequenced.

Multiple isolates from individual habitats (warthog burrow and pig pens) were found to be identical across the sequenced portion of the gene resulting in 19 unique isolates of ASFV. Representatives of each unique sequence were compared to that of 36 reference viruses representing all 23 previously described *p72* genotypes ((I‐XXIII); Table 2).

**Figure 2:**
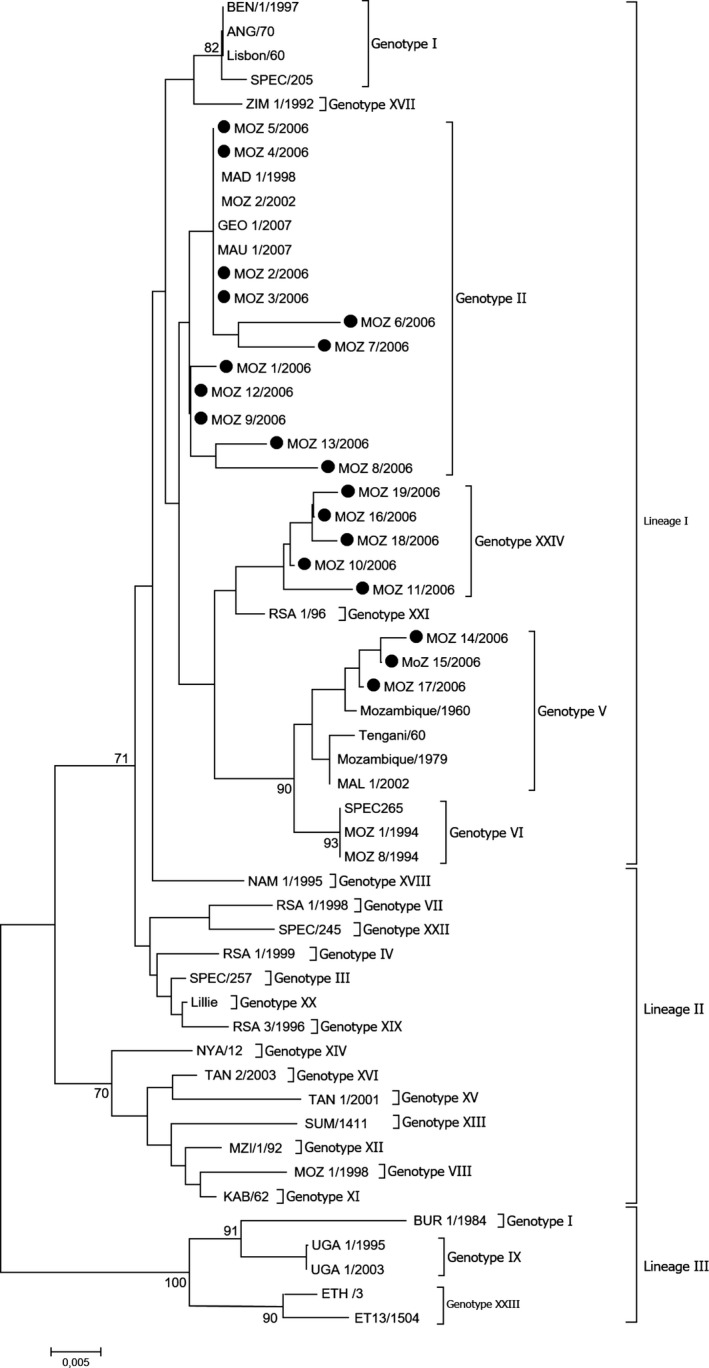
Phylogenetic relationship of 55 ASF virus isolates based on p72 gene sequences. The evolutionary history was inferred using the Neighbour‐joining method. The percentage of replicate trees in which the associated taxa clustered together in the bootstrap test (1,000 replicates) is shown next to the branches. Isolates characterized in this study are marked with closed circles

The viruses clustered into three distinct evolutionary lineages (Figure 2), each one confined to a broad geographical area (Boshoff, Bastos, Gerber, & Vosloo, 2007) and supported by high bootstrap values (69% to 100%). Lineage I comprised 13 genotypes associated with viruses from West and southern Africa, while lineage II consisted of viruses from East Africa and lineage III consisted of viruses from the Great Lakes Region of East and Central Africa (Achenbach et al., 2016; Bastos et al., 2003, 2004; Lubisi et al., 2007). Most of the viruses historically isolated in Mozambique were grouped in lineage I, except for MOZ 1/1998, which was classified as belonging to genotype VIII of lineage II (Bastos et al., 2003).

The three lineages were further divided into 23 previously described genotypes as well as the novel 24th genotype (I‐XXIV). The ASFV isolates from this study clustered into three genotypes within lineage I (Figure 2). Eleven of the 19 isolates clustered within genotype II with four being 100% homologous over the *B646L* gene region sequenced and indistinguishable from other viruses previously classified as belonging to genotype II (MAD 1/1998, MOZ 2/2002, MAU1/2007 and Geo 1/2007) (Bastos et al., 2003; Lubisi, Bastos, Dwarka, & Vosloo, 2005; Lubisi et al., 2009; Rowlands et al., 2008). The remaining eight viruses were all genetically distinct and differed by between 1.25% and 2.75% at nucleotide level from the other Mozambique viruses within genotype II. Three viruses from this study clustered within genotype V and were genetically related (nucleotide identity >97%) to viruses previously isolated from warthogs in Malawi (Tengani/60) and ASF outbreaks in Mozambique (Mozambique/1960, Mozambique/1979) and Malawi (Mal 1/2002) (Bastos et al., 2003, 2004; Lubisi et al., 2005). The remaining five isolates grouped as a separate cluster and new genotype, defined as genotype XXIV (bootstrap support of 75% and <3% nucleotide divergence). This new genotype included viruses from domestic pig pens (MOZ 18/2006 and MOZ 19/2006) as well as warthog burrows (MOZ 10/2006, MOZ11/2006 and MOZ 16/2006) (Figure 2), illustrating a potential transmission between the domestic and the sylvatic cycle.

Although the clustering of the isolates based on the *CP204L* (*p30*) and *E138L* (*p54*) genes was similar to that generated using *p72*, individual *p30* or *p54* genotypes were generally incongruent using the criteria of pairwise difference of 0.96% and >80% bootstrap support to define a genotype (data not shown). This suggests that the genetic diversity within these gene regions is not sufficient to infer the phylogenetic or evolutionary relatedness of ASF viruses.

### 3.4 Comparison of ASFV isolates from the study area and southern Africa using the B602L gene

The number of amino acid (aa) tandem repeats in the *B602L* gene region is a highly variable genetic marker and was used to differentiate closely related *p72* genotypes. The *B602L* gene of 16 of the 19 ASFV strains collected in this study was successfully sequenced and was compared to six additional closely related viruses within each of the respective *p72* genotypes (Table 3). Seventeen different aa tetramers were found: B = CADT; N = NVDT/NVGT; D = CASM; A = CAST; L = CTST; H = NEDT; P = NADT; S = SAST; O = NASI; F = NAST; Q = NADI; V = NANT; M = NANI; T = NVNT; C = GAST; K = CANT. The viruses were classified into eight subgroups namely XXI, XXIa, XXIb, XXIc, XXXII, XXXIII, XXXIIIa and XXXIIIb (Table 3). Additionally, the isolates from this study were classified into six of the eight subgroups, based on their length and sequence of aa tetramer repeats (Table 3). The results indicated a high level of genetic variation for the viruses from Mozambique.

All isolates that clustered in *p72* genotype II belonged to CVR subgroup XXXII. However, the CVR aa sequence of MOZ 1/2006 differed substantively from that of the other viruses contained in *p72* genotype II and was found to be identical to a virus within genotype V (MOZ 15/2006) isolated from a pool of N4 nymphs from the GNP. Three ASFV that had been described from outbreaks in Madagascar (1998), Mauritius (2007) and Mozambique (2002) also fell into CVR subgroup XXXII, with 10 aa repeats, indicating the close relationship between these viruses. The genetic variation within genotype II is surprisingly low considering that these viruses were sampled over an 18‐year period from both domestic pigs from previous investigations and the argasid vector from this study.

Three of the isolates from the novel p72 genotype XXIV had their own CVR subgroups (XXXIII, XXXIIIa and XXXIIIb) forming unique aa tandem repeats and sequences that have not been described before.

In contrast to genotype II, viruses within genotype V displayed a high level of within‐genotype genetic variation. Seven viruses included in the analysis were classified into four distinct CVR subgroups (XXI, XXIa, XXIb and XXIc). This included historical viruses implicated in ASF outbreaks in Mozambique and Malawi (Bastos et al., 2004; Nix et al., 2006), as well as four contemporary virus strains isolated from ticks collected in the GNP (Table 3).

Similar to viruses in genotype V, isolates belonging to the newly identified *p72* genotype XXIV displayed a high level of genetic diversity. Three unique CVR subgroups (XXXI, XXXIa and XXXIb) were identified, each represented by a single virus. Interestingly, the CVR sequence of MOZ 16/2006 differed from that of subgroup XXIb at only two of the 12 tetramers that make up the sequences, despite the *p72* sequences of these viruses sharing <97% identity.

## 4 DISCUSSION

Despite the suspected importance of a sylvatic cycle of ASFV in sub‐Saharan Africa (Bastos et al., 2009; Gallardo et al., 2009), information about the ecology of ASF and its wild hosts remains scarce. This study was able to confirm the occurrence and genetic heterogeneity of ASFV isolated from infected soft ticks collected from warthog burrows and domestic pig pens at the wildlife/domestic interface of the GNP in central Mozambique.

African swine fever was first diagnosed in 1960 in the central western part of Mozambique adjacent to the Malawi border (Haresnape, Lungu, & Mamu, 1985). Since then, repeated outbreaks reported in the northern and central provinces have been suspected to originate from spill‐overs of a potential sylvatic cycle maintained by free‐ranging warthogs and ticks in infected wildlife areas such as the GNP (Abreu, Valadão, Limpo Serra, Ornelas, & Montenegro, 1962). The disease was only reported in the southern provinces of the country three decades later, in 1994. Since then, sporadic outbreaks have been more frequently described in endemic areas, where there is a high density of small‐scale farmers implementing limited biosecurity measures linked with important trade movements of live pigs (Penrith et al., 2007). In certain areas, the virus is suspected to circulate primarily within a domestic pig‐to‐pig cycle, in which the involvement of ticks could be potentially important but was never confirmed (Matos et al., 2011; Penrith et al., 2004). Due to the high number of pigs (estimated in 1.3 million) kept by small‐scale farmers and the large diversity of ASF strains maintained in the sylvatic cycle, Mozambique seems to act as an important reservoir of ASFV for other countries in the region and beyond. Although the sampling in this study was spatially limited, the prevalence of soft tick infestation found in warthog burrows was higher (90%) than the one reported in other wildlife areas assessed in East and southern Africa (Plowright et al., 1994; Wilkinson, Pegram, Perry, Lemche, & Schels, 1988).

Despite the limitations of our sampling approach in domestic pig pens from villages located in the BZ, 200 tick specimens and two ASF virus isolates could be recovered to characterize ASFV presence in this type of habitat. Based on sequencing of the tick 16S rDNA gene, tick populations found in wild and domestic habitats were distinct as suggested by Walton's classification (Walton, 1962, 1979) with *O. porcinus domesticus* found in pig pens and *O. porcinus porcinus* found in warthog burrows suggesting an absence of exchange between tick populations. Nevertheless, there was a high level of homology between the isolates found in the ticks collected from domestic habitats (MOZ 18 and MOZ 19/2006) and some of those found in warthog burrows (MOZ 16/2006, MOZ 10/2006), providing evidence that a certain level of virus transmission occurs at the wild domestic suid interface.

In a study in the same area of the GNP interface, the presence of antibodies against soft tick salivary glands was found in domestic pigs. A significantly higher proportion of domestic pigs were positive to anti‐tick antibodies in areas closer to the GNP, suggesting that proximity to this wildlife area may contribute to a higher exposure of the pig population to *Ornithodoros* spp. bites than those distant from the GNP (Quembo et al., 2014).

The genetic analysis of ASFV in the study area suggested potential epidemiological links between the sylvatic cycle of GNP and outbreaks reported elsewhere (Boshoff et al., 2007; Gallardo et al., 2014; Haresnape & Mamu, 1986; Lubisi et al., 2007). Genetic characterization of viruses belonging to the *p72* genotype II indicated a high level of homology between newly characterized viruses and those that have caused outbreaks in Mozambique (MOZ 2/2002), and during the last decade outside Africa (Madagascar (MAD/1/98), Mauritius (MAU1/2007) (Bastos et al., 2003, 2004; Lubisi et al., 2005, 2009) and eastern Europe (Gallardo et al., 2014; Rowlands et al., 2008). This close homology was further supported by findings based on other gene regions such as the *p30*,* p54* and *B602L,* indicating that these viruses were genetically similar and probably from a common wild host origin.

Interestingly, ASFV isolates belonging to genotype V were reported in warthogs and in domestic pigs from Mozambique and Malawi more than 50 years ago (Abreu et al., 1962; Bastos et al., 2004), indicating the capacity of some genotypes to be maintained for at least half a century in the sylvatic cycle.

Of importance was the finding of a novel genotype reported for the first time in soft ticks (XXIV), highlighting once more the diversity of ASFV variants found in the sylvatic cycle. Most new viruses are only characterized once they reach domestic pigs and cause outbreaks. Therefore, there is an urgent need to investigate, quantify and characterize ASFV variants present in the sylvatic cycle in sub‐Saharan Africa, in order to better understand and monitor virus variability and diversity and explore potential strains that could contribute to the development of vaccine. Interestingly, based on p72/CVR sequence results or the lengths of the CVR amplicons, there was no indication that our burrow samples contained more than one genetic variant of the virus. However, further research with specific methods (cf. cloning virus isolates before PCR amplification) should be undertaken in future studies to assess ASFV genetic variability per burrow and to understand potential ecological and biological drivers affecting this variability.

In conclusion, the results obtained in this study suggest that soft ticks found in natural and domestic habitats at the GNP interface act as a permanent source of different strains of ASFV for pigs reared in free‐ranging conditions in that area. The high infestation rates and genetic diversity of viruses found in those ticks were pronounced and included previously identified genotypes (II, V) and but also a new genotype identified for the first time (XXIV) in this study. These results highlight the epidemiological importance of the sylvatic cycle in harbouring and disseminating new and existing virus strains in the Mozambican pig value chain. Moreover, the recurrent emergence of genotype II ASF outbreaks outside of the African continent during the last decade genetically linked with isolates from Mozambique (including the one currently circulating in eastern Europe) highlights the importance and the need to further investigate the characteristics, distribution and diversity of the ASFV maintained within wild hosts in East and southern Africa and the transmission patterns and pathways followed by the virus to spread to the local domestic cycle and more globally into the international pig and pork product value chains.
